# Researchers’ views of risk of bias in cluster randomised trials: a qualitative interview study

**DOI:** 10.1136/bmjopen-2025-103091

**Published:** 2025-11-05

**Authors:** Christina L Easter, Caroline Kristunas, Sheila Greenfield, Karla Hemming

**Affiliations:** 1 Department of Applied Health Sciences, University of Birmingham, Birmingham, UK; 2 Cancer UK Clinical Trials Unit, University of Birmingham, Birmingham, UK

**Keywords:** QUALITATIVE RESEARCH, Research Design, STATISTICS & RESEARCH METHODS

## Abstract

**Abstract:**

**Objectives:**

Cluster randomised trials (CRTs) can be at risk of bias driven by differential identification and recruitment of participants across treatments, posing a threat to the validity of findings. We explored the awareness and importance, among CRT researchers, of the recommended bias mitigation measures.

**Design:**

Qualitative interview study using semistructured interviews.

**Participants:**

Participants were researchers involved in conducting CRTs, including investigators, statisticians and trial coordinators. 24 participants, including statisticians (n=13, 54.2%), clinical investigators (n=9, 37.5%) and trial coordinators (n=2, 8.3%), were interviewed; with representation from the UK (n=10, 41.7%), Australia (n=5, 20.8%) and the USA (n=4, 16.7%).

**Results:**

Participants exhibited differing levels of knowledge related to biases. Some participants demonstrated high levels of knowledge, but we also identified prevalent misconceptions, with some evidence of superficial knowledge. While some participants worked in collaborative teams, other teams’ responsibilities were delineated, and this impacted on how knowledge of biases was shared and acted on. Logistical and practical issues could prevent known solutions to mitigate biases being implemented. Biases also manifested because of a perception from participant recruiters that the purpose of research is for participant benefit rather than producing generalisable knowledge; and a normalisation or expectation that CRTs produce a lower level of evidence.

**Conclusions:**

There is an urgent need to ensure that CRTs are free from risks of bias. Mitigation measures are either not known, not practical or unconsciously subverted. More education and collaborative working might help. Preventing subconscious bias during participant recruitment and dispelling the myth that CRTs produce lower levels of evidence would require a change in culture.

**Strengths and limitations of this study**

- Conducting a qualitative interview study provided an in-depth insight into researcher’s views.
- Use of online video calling (eg, Zoom) allowed the inclusion of participants from multiple countries.
- Recruitment from low-income and middle-income countries was low even with the availability of video calling.
- A change to our planned recruitment strategy to a snowballing approach very likely skewed our sample towards researchers with a particular interest in cluster trials.

## Introduction

 Typically, clinical trials are conducted to assess an intervention against a control condition. Individually randomised trials randomly allocate the participants to these different treatments. In contrast, cluster randomised trials (CRTs) randomise groups of participants such as wards, schools or social groups.^1 2^ The cluster randomised design uniquely allows evaluations of interventions that are delivered at the level of the cluster, but they are also used to simplify trial processes, reduce the risk of contamination or to determine the effect of interventions when embedded in usual care.^3^ As a result, the use of the CRT design has been increasing over recent years.^4^

However, for interventions that are delivered directly to the individual (referred to as an individual-level intervention), such as a drug, both cluster and individual randomisation are theoretically possible. Unfortunately, when the CRT is used to evaluate individual-level interventions, biases can arise through differential identification and recruitment of participants across treatments, resulting from knowledge of the treatment allocation at the time of participant recruitment.^5^ These risks pose a serious threat to the validity of the findings from CRTs.^5^,^9^

While there are recommendations to help investigators mitigate these risks of bias (including using individual randomisation where possible; recruiting participants before randomisation; blinding treatments or recruitment by someone blind to the allocation), these recommendations are rarely followed.^10^ Consequently, there is an important need to understand why this is happening, so as to actively identify solutions that are amenable in practice. To our knowledge, this has not been explored before.

We carried out semistructured interviews with researchers involved in CRTs to understand and explore (1) the rationale behind using the cluster randomised design when the intervention is delivered at the level of the individual; the awareness and importance of (2) recruiting participants before randomisation where possible; (3) the blinding of those who identify and recruit participants and (4) the barriers and facilitators for a successful uptake of these recommendations.

## Methods

A brief summary is outlined below with the full details available in the published protocol and associated supplementary material.^11^

### Participants

We invited researchers who had been involved in conducting CRTs, including investigators, statisticians and trial coordinators. The initial sampling frame consisted of authors from a published review of CRTs evaluating individual-level interventions, ordered by year of publication^10^; and was subsequently extended using a snowball approach.^12^ Characteristics of participants (including professional/research role, country, years in research, number of CRTs involved with) were monitored throughout using a questionnaire to ensure inclusion of a diverse range of researchers.^11^

### Recruitment

**Figure 1:**
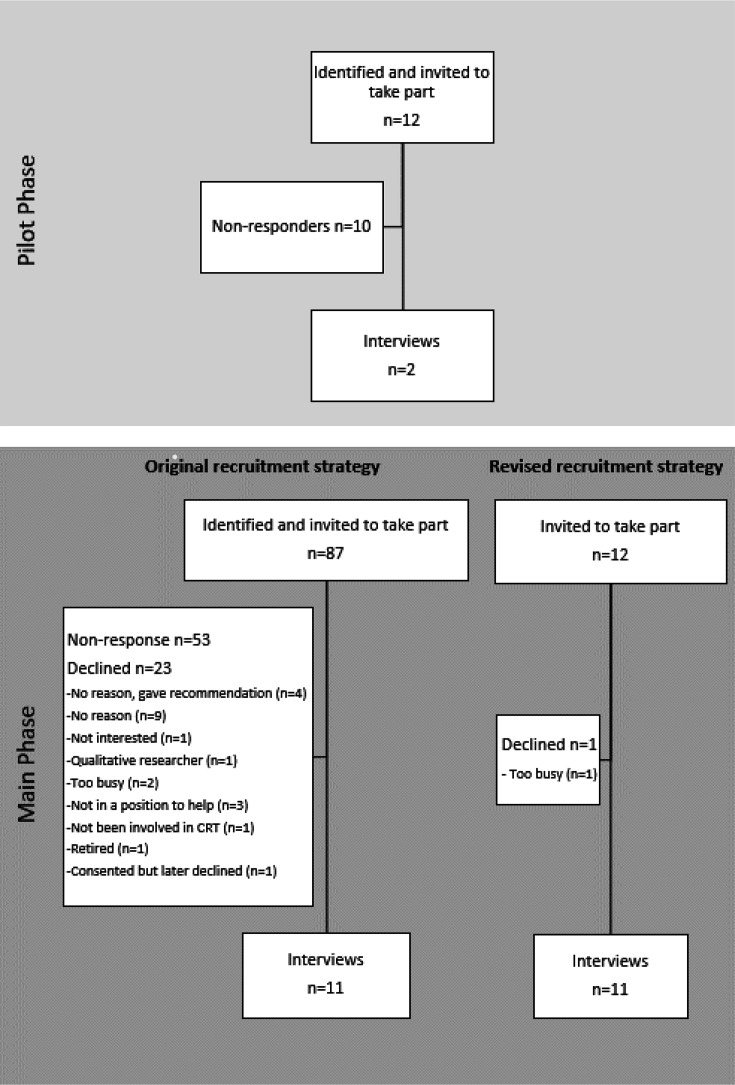
Flow diagram for recruiting participants for both the pilot and main phase of this study. CRT, cluster randomised trial.

Potential participants were contacted via email. For the pilot phase, researchers were contacted in batches of 5–6; and in the main phase in batches of 10 on a fortnightly basis. The list of identified researchers was exhausted before we had reached saturation point, that is, no new further information was being discussed within the interviews.^13^ The recruitment method was subsequently modified to incorporate a snowball approach by contacting prominent researchers within the CRT community, asking that they forward the invitation to potential participants (onlinesupplemental materials 1  2). This overall approach yielded a diverse range of participants and saturation was considered to have been reached at 24 interviews (figure 1, table 1).

**Table 1:** Characteristics of participants who completed an interview

Characteristics	N=24
Positions held when conducting CRTs	
Investigator*	9 (37.50)
Statistician	13 (54.17)
Trial coordinator	2 (8.33)
Number of CRTs involved in	
Less than 5	7 (29.17)
5 or more	17 (70.83)
Country	
UK	10 (41.67)
Australia	5 (20.83)
USA	4 (16.67)
Canada	2 (8.33)
Pakistan†	2 (8.33)
South Africa	1 (4.17)
Sex	
Female	12 (50.00)
Male	12 (50.00)
Years in a researcher role	
Median (IQR)	17.6 (11.5–21.5)
Specific CRT experience:	
Previous involvement of designing CRTs	23 (95.83)
Previous TSC involvement	4 (16.67)
Previous DMC involvement	4 (16.67)
Self-reported role‡	
Professor	9 (37.50)
Associate professor	1 (4.17)
Senior lecturer	2 (8.33)
Statistician	14 (58.33)
Policy advisor	1 (4.17)
Head or director of a research group	4 (16.67)
Senior research fellow assistant	2 (8.33)
Global health academic	1 (4.17)
Clinician	1 (4.17)

All summary statistics are displayed as frequency (%) unless otherwise stated.

* This includes principal investigators (PI), co-PIs, chief investigators, clinical investigators, epidemiologists and research fellows.

† 1 participant conducts research in Pakistan but lives elsewhere.

‡ n=24 with non-mutually exclusive categories.

CRT, cluster randomised trial; DMC, Data Monitoring Committee; TSC, Trial Steering Committee.

### Data collection

#### Interview material

The topic guide was devised to align with the study objectives and covered recruitment, selection bias and design justification, which was centred on a fictional illustrative case study to avoid any apparent criticism of researchers’ own trials. This case study illustrated a CRT evaluating an individual-level intervention without blinding, where participants were recruited postrandomisation, and a subsequent comparison of participant characteristics across treatments showed substantial imbalance (online supplemental file 3). The topic guide was developed and tested in practice interviews before starting the formal pilot phase. Feedback from the pilot participants was documented and some small amendments were made to the final topic guide (online supplemental file 4).

#### Interview process

All interviews were conducted and recorded in Zoom solely by CLE (female PhD statistician) making field and reflective notes^14^,^16^ and transcribed verbatim (CLE). Debrief meetings were held after the first five interviews to allow the research team to reflect on challenges. Transcripts were anonymised and any identifying information redacted. One-page summaries were created using a modification of the ‘one-sheet of paper’ method.^17^ These summaries were shared with the interviewees, who were invited to make comments or alterations as they saw appropriate.^18^

### Data analysis

Pilot and main phase participant transcripts were analysed together using NVivo for data management. The research team discussed the content, both across and within interviews, enabling real-time adaptation throughout the interview process, making some small additional changes to the topic guide. Monitoring the content of the interviews allowed us to determine when the saturation point had been reached.

On completion of the interviews, an outline of the content across the interviews was constructed initially by CLE and KH with input from all members of the research team. A realist approach was adopted throughout, with thematic analysis using an inductive approach,^19^ allowing emergent themes to develop without using preconceived themes to drive the assessment. All research team members independently viewed a selection of transcripts and met to discuss the findings to allow individual perspectives to aid theme development. An overall consensus was reached through these discussions on the themes and subthemes that were generated from the data.

## Results

### Participants

Eight practice interviews were completed with University of Birmingham researchers involved in CRTs but were not included in the formal analysis. 12 researchers were invited to participate in the pilot interviews with two providing written consent. For the main phase, 99 researchers were invited and 22 gave written consent (figure 1). Over time, there was a noticeable decline in response to the email invitation. Those that declined participation mainly gave no reason (n=9, 39.1%), although some provided details of other potential participants (n=4, 17.4%). The pilot and main phase interviews took place between January and September 2022 with a median duration of around 50 min (IQR: 44, 58). One-page summaries largely remained unchanged on participant review. However, a few participants provided further clarification which was incorporated into the transcripts.

24 participants (evenly distributed by gender) took part in the interviews across the pilot and main phase (table 1). The participants included statisticians (n=13, 54.2%); clinical investigators (henceforth referred to as ‘investigators’), who include principal investigators as well as coinvestigators (n=9, 37.5%); and trial coordinators (n=2, 8.3%). The majority of participants were from the UK (n=10, 41.7%), Australia (n=5, 20.8%) and USA (n=4, 16.7%). The median length of time in research was 17.6 years (range from 4 to 40 years). Nearly all participants reported having an active role in designing CRTs (n=23, 95.8%) and a few reported being part of Trial Steering Committees or Data Monitoring Committees for a CRT (n=4, 16.7%).

### Main themes

Four main themes were identified: knowledge and misconceptions; alliances and collaborations; logistical and practical issues; and unconscious biases.

#### Knowledge and misconceptions (theme 1)

Throughout the interviews, it was clear that there were differing levels of knowledge of risks of bias in cluster trials. We categorised the different types of knowledge as (1) high levels of knowledge (in relation to these biases); (2) misconceptions (where participants held a belief that was at odds with good practice guidelines) and (3) superficial knowledge (where participants used appropriate terms but without a clear demonstration of understanding). These are expanded on in table 2.

**Table 2:** Theme 1: knowledge and misconceptions

Subtheme	Explanation	Quote
High-level knowledge (intrinsic facilitator)	Some participants had a clear understanding that implementing a cluster trial in a particular way could introduce risks of bias, that would ultimately down-grade the quality of the study.	“[Imbalances in a baseline table] downgrade(s)the quality of the evidence, you've lost that true randomisation, I mean even grade in the sense of the GRADE(Grading of Recommendations Assessment, Development, and Evaluation)criteria for assessing research, you start off a randomised trial at quite a high level of evidence, but as soon as you start to identify risks of bias, you can down-GRADE that evidence” (Professor of Medical statistics, UK, 12yrs research experience)
	Alternatively, for example, they demonstrated a deep level of knowledge around the trade-offs between contamination within an individually randomised trial and other biases that can arise in a cluster trial.	“I guess we have in mind that on an individually randomised trial could be too biased, because of contamination. But as I mentioned earlier, you know there is a trade-off there. Uhm, it could be for other reasons that a cluster randomised trial would also be biased and therefore you're choosing you know which one is less biased. And sometimes another factor to think of is that with contamination that’s normally a bias in a known direction, it’s biased towards the null whereas these other biases that can affect a cluster randomised trial, sometimes we don't know the direction of those biases. So it’s not only trading off the magnitude of the bias, but you could think that contamination is a slightly more benign bias because it’s in a known direction generally.” (Professor of Statistics, UK, 20yrs research experience)
	Others underscored the practical importance of this level of knowledge or expertise.	“I guess all those experiences did impact on how we designed Trial B because of the knowledge from the, from that first [CRT] trial” (Senior lecturer in Statistics, UK, 20yrs research experience)
	However, it was also recognised that even expert advice or knowledge might not be sufficient to mitigate all biases.	“there will have been discussions presumably at the design stage of this trial about how it should work and they would have been independent external advice about how it should work, you know as with any trial, and it would have been done with the best intentions.”(Professor of Medical statistics, UK, 12yrs research experience)
Misunderstandings or misconceptions (intrinsic barrier)	Other participants demonstrated a lack of knowledge or misunderstanding around the theory of randomisation, not just related to cluster trials but sometimes to trials more generally. Various misconceptions were identified. For example, some participants suggested that covariate adjustment could mitigate the impact of any biases induced by post randomisation recruitment biases (while covariate adjustment has an important role, recruitment biases render the study more like an observational study than a trial^7^).	“but I would adjust for factors which erm were deemed to be imbalanced and the way we would usually do that is before we looked at the data we would look at the baseline factors and decide which ones, we would adjust for if they showed a substantial imbalance at baseline” (Professor of Epidemiology and Statistician, UK, 25yrs research experience) “cluster randomised trials have far smaller sample sizes, so you may well get imbalance, but you can adjust” (Group CEO of an institute, South Africa, 31yrs research experience)
	Others questioned the recommendation of recruiting participants before randomisation (even when this was a feasible approach).	“if all of the participants are already recruited and then you randomise them? I think, no, I think we should randomise it before recruiting them into the study.” (Senior research fellow, Pakistan, 4yrs research experience)
	While the suggestions above were likely made in a naïve way, adding to the complexity, there was recognition by some that these tests can have a place in CRTs with post randomisation recruitment.	“Statisticians normally advise against doing hypothesis tests to compare characteristics in randomised arms because if the randomisation has worked properly, there there’s no point working out a P value to decide if one arm looks different to another because they've been randomised. I mean, that’s not quite true always because if there was some systematic bias at play, the hypothesis test and the P value could measure the evidence for that systematic bias.” (Professor of Medical statistics, UK, 12yrs research experience)
Superficial knowledge (intrinsic barrier)	Some participants used technical terms, and thus appeared to be demonstrating a high level of knowledge, but in a way that seemed to be at odds with the typical meaning of the term. This often went hand in hand with confidence. One of the main occurrences of this was the use of ‘selection bias’, which was frequently used in a way that suggested a misunderstanding of its meaning in cluster trials (in cluster trials the term ‘selection bias’ is used to refer to differential identification and/or recruitment across study arms (ie, internal validity), and not perhaps the more common understanding of the term which is about generalisability.^7^	“The type of participants who would agree to be in a study might be different in one group vs another group, and might be different between those who do, and those who don't participate.” (Professor of Biostatistics, Canada, 20yrs research experience) (in response to a question asking for the meaning of the term selection bias in this context)
	Another participant acknowledged that these terms did not always have a consistent meaning and moreover, used terms in a potentially confusing way. In this instance suggesting the term selection bias might be to do with a (inoculant) chance imbalance by the use of the word residual.	“That is what I call selection bias and different people call it, it differently, you know people call you know residual confounding.” (Associate Professor of Epidemiology and Statistician, US, 12yrs research experience)
	Others suggested that the problems arising because of unblinded post randomisation recruitment could be fixed by restricting the randomisation (restricted randomisation helps mitigate large chance imbalances but does not prevent post randomisation recruitment biases^7^).	“there’s other methods you can use, minimization there’s all these techniques you can do to minimize different, you know apriori differences in clusters that could result in something like this.” [referring to participant imbalances across arms in the baseline table] (Professor of Medicine, US, 28yrs research experience)

CRT, cluster randomised trial.

#### Alliances and collaborations (theme 2)

Participants appeared to hold differing ideas around how teams and responsibilities should be aligned. Sometimes we identified very collaborative ways of working, but other times participants appeared to want to carefully differentiate different responsibilities to different roles that seemed to suggest a disjointed way of working. We categorised these different views around responsibility into (1) collaborative teams (where members shared the same goal of working together as a team) and (2) delineating responsibilities (where members indicated a preference for aligning different responsibilities to different roles). Table 3 details quotes and explanations for these subthemes.

**Table 3:** Theme 2: alliances and collaborations

Subtheme	Explanation	Quote(s)
Collaborative teams (intrinsic facilitator)	Some participants (both investigators and statisticians) expressed a clear preference for collaborative ways of working. When interviewing investigators this often manifested as an investigator who recognised the limits of their knowledge of cluster trials and would seek expert support and advice to proceed with designing their study.	“I had a fair understanding of cluster randomised trial and I understood how it would work and why we needed it. But I had a clinical trials unit and a particular one, you know one particular person who is an expert, who was a statistician and he guided me along the way, that’s really what you need with something like this, you can't you know, you can't do it correctly without people like that.” (Research group lead, UK, 17yrs research experience) “Gosh it’s hard to kind of tease apart the factors isn't it really, to make the decision. I guess it’s why you use a clinical trials unit.” (Professor of Behavioural Medicine, UK, 23yrs research experience) “I would ask you the statistician how do I deal with that” (Infectious Disease Clinical Researcher, Australia, 5yrs research experience)
	These collaborations extended within the team more generally, not necessarily just between the statistician and investigator.	“PI’s right hand man, well right hand woman… Well the PIs come up with bright ideas, let’s do this let’s do that let’s do the other and then I've come up to, oh no we can't do that because of this you know, I scaled them down a bit.” (Senior Research Associate, UK, 15yrs research experience)
	We also observed this shared way of working among statisticians where it manifested as a desire to engage in discussions to ensure that their knowledge was considered in the key moments that would impact the designing of the trial. In particular we saw that this level of engagement could be especially important when it came to choosing if the CRT was the right study design.	“I would have tried to avoid a cluster trial unless it was absolutely necessary and I can’t see anything here [referring to the case study], and I would talk with the research team as a statistician. Check whether there is a valid reason for a cluster trial. If I don't think there is and if I, I would certainly engage with them in discussions about contamination, about the timing and process of recruitment, so sticking to contamination issue, I will see whether it is feasible and appropriate, given the intervention for somebody else to deliver the intervention and, if so, whether the GP really needed to know that their patient had received this leaflet.” (Senior lecturer in Statistics, UK, 20yrs research experience)
Delineating responsibilities (intrinsic barrier)	Other participants appeared to want to carefully differentiate different responsibilities to different roles. To this end, the responsibility for different aspects of designing the trial were sometimes redirected to other researchers in the team. For investigators, this, for example, might manifest as expecting that the study statistician could fix issues at the time of the statistical analysis (despite it being much better to fix these issues at the design stage).	“there’s a slight increase in males in one arm, in the intervention arm, so you, a statistician would have to look at this and see you know, maybe in your analytic[al] plan you adjust for gender.” (Professor of Medicine, US, 28yrs research experience) “what the statisticians always do is they adjust for baseline, I don’t know what, they must adjust for other things, must they?” (Senior Research Associate, UK, 15yrs research experience) “And then you have to work out, I would ask you the statistician. How do I deal with that, my ICC is going through the roof, don't know what to do.” (Infectious Disease Clinical Researcher, Australia, 5yrs research experience) (In response to seeing baseline differences)
	Others suggested a more tokenistic collaborative way of working.	“at one point, we spoke to the clinical trials unit. But it was just the statistician and somebody who ran the GP network” (Professor of Behavioural Medicine, UK, 23yrs research experience)
	For statisticians, the delineation of tasks manifested as a belief that the statistician role might not include mitigating bias.	“so as a statistician like maybe I shouldn’t be so upset about bias” (Director of clinical research group and Statistician, US, 20yrs research experience) “as a statistician as well, you can work around it in some ways, like if you don’t get the, you know you can adjust in the analysis in some ways.” (Biostatistician, Australia, 21yrs research experience)
	Or, describing the role of the statistician in a very limited way.	“as a statistician with cluster trials is working out the most appropriate way of calculating the required sample size in the first place, the most appropriate measure techniques for analysis as well.” (Post-doctoral statistician, UK, 5.5yrs research experience)

CRT, cluster randomised trial.

#### Logistical and practical issues (theme 3)

While the interview focused around a simplistic case study, participants shared their experience of logistical and practical issues encountered in attempting to implement recommended methods to mitigate bias. We categorised this theme into (1) theory-practice disconnect (whereby recommendations for good practice simply did not work in the real-world); (2) the feasibility of implementation (recommendations for good practice might work but only at considerable cost) and (3) grass-roots level solutions (whereby participants came up with their own recommendations). Table 4 displays further details regarding these subthemes.

**Table 4:** Theme 3: logistical and practical issues

Subtheme	Explanation	Quote
Theory-Practice disconnect (extrinsic barrier)	Many participants expressed views that in practical settings it was simply not feasible to follow some of the known recommendations for preventing biases, demonstrating a discourse between theoretical methods and practicalities of conducting a cluster trial. For example, some participants commented on how in practice it is not possible to recruit participants then randomise clusters.	“it would just be so logistically difficult. You know it would help if they'd all done a baseline before the randomisation. But then it would take so long to get, you know, between that baseline and when they got the materials [intervention] it just doesn’t make sense in light of the intervention. Mostly it’s because patients are not available every day in a Doctor’s office.” (Professor of Statistics, US, 40yrs research experience) “the patients could be approached before the randomisation as well. I've never been in a fortunate position of that ever-taken place, but that would be, that would be good.” (Professor and the head of a biostatistics unit, Australia, 29yrs research experience)
	Moreover, others revealed that despite the trial team having knowledge of these recommendations in settings where they had been impossible to follow, they had nonetheless come under considerable pressure to implement them.	“one of our study went to an ethics committee and they wanted us to recruit everybody before we deliver, we're revealed the randomisation and they could not just, they would, they couldn't understand the reasons why we couldn’t do this, and we argued and argued, and it was agreed that the Chair, I was able to have a discussion with the Chair, because they weren't getting, so [getting] fed up with this discussion, I said you can't recruit 40 patients in a practice and then deliver the intervention to all of them at one go.” (Senior Research Associate, UK, 15yrs research experience)
	Others noted how blinding interventions (one of the recommendations) is often not aligned with the types of interventions evaluated in cluster trials.	“I guess the thing with a behavioural intervention like this is that, sometimes it’s difficult to blind people discussing it. So, could it be possible, introduced biases I mean sure, yes, of course, that’s why you want, it’s like a double blinded study, or a double blinded placebo study to really get rid of any kind of bias.” (Senior Policy Advisor, Canada, 15yrs research experience)
	Or, they simply struggled in trying to identify a solution that might work in either the case study or in one of their own trials.	“there’s not always a nice solution” (Head of school, Australia, 15yrs research experience) “I can't think of a, um solution” (Senior lecturer in translational research, UK, 9yrs research experience)
	One participant even stated that local laws make it impossible for researchers to follow one of the recommendations (recruitment by anyone other than the doctor can be illegal in Canada).	“in our context no investigator or study personnel now would have direct first contact with patients” (Professor of Biostatistics, Canada, 20yrs research experience)
Feasibility of implementation (extrinsic barrier)	While in some settings it might simply not be feasible to follow some of the known recommendations for preventing biases, in other settings, participants expressed a view that it might be feasible but challenging. These challenges often related to expense and logistical complications of implementing solutions. For example, some participants spoke about the difficulties induced by the need to rely on external partners goodwill.	“So there could be logistical headaches. Uhm, if it’s got to be done in real time, I think, uh, if you could identify all the patients in advance, then you could kind of send the list to the CTU and the allocations kind of come back, but if it’s going to be done in real time then that could be logistically difficult, could be disruptive to appointments, GPs might just refuse to do it.” (Professor of Statistics, UK, 20yrs research experience)
	Others alluded to the additional costs (that might not have been factored into the grant application).	“I suppose, ideally practically that that person coming in, would be blinded to the allocation and that that could be the allocation of the GP practice and that could be difficult. Erm, and then of course there’s the additional expense associated with getting someone involved to, that is external to each practice.” (Associate Professor of Biostatistics, Australia, 8yrs research experience)
	Others were less specific, but expressed similar concerns.	“So it all comes down to the logistics and whether it’s practical and the cost involved” (Biostatistician, Australia, 21yrs research experience)
Grass-roots level solutions (internal facilitator)	Participants offered their own thoughts of how these trials might be conducted to reduce these risks of bias. A wide range of practical solutions were suggested. For example, it was suggested that when recruiting participants into a study, having an objective inclusion criterion might reduce the risk of differential inclusion.	“if we make the criteria’s objective and the assessment team is blind and only GPs are not blind, that’s what we discussed right. So yeah that will also create less room for GPs to introduce their personal or selection bias to pick any participant or not pick any participant.” (Senior research fellow, Pakistan, 4yrs research experience)
	Others suggested the use of electronic records to monitor if all eligible participants have been invited to take part in the trial.	“So I guess at the start of it you'd ask the GP to send his list of people [patients] erm unless, of course, you can do everything through any EHR [Electronic Health record] which is more and more how things are done, electronic medical record.” (Professor of Medicine, US, 28yrs research experience) “I think an alternative worth considering, would be to run the electronic searches in the practices, the research team runs the searches, the research team sends out the invitation” (Senior Research Associate, UK, 15yrs research experience)
	Other suggestions included the use of pilot studies or including homogeneous clusters might also be ways of reducing biases. It was also noted that (where ethically appropriate) waiver of individual participant consent would mitigate these biases.	“in the case where it’s much easier to allocate everyone in the same hospital on the same unit to the same intervention just in terms of doing it, then they're willing to make a case that the public good for this outweighs the erm, oh they will be going for a waiver of consent in this so that with a waiver of consent they can justify, they try and justify the waiver consent, saying that there are no real harms that could come from this, because we know both products, both interventions are safe and the public health good or the scientific knowledge that could come out of this leading from a trial that wouldn't be possible individually randomised because just too hard to get consent from patients, the waiver of the, the benefit to the public outweighs the I suppose the ethical considerations to the individual to provide consent” (Professor and the head of a biostatistics unit, Australia, 29yrs research experience)

#### Unconscious biases (theme 4)

Participants also either demonstrated or reflected on prevalent unconscious biases. We categorised these subthemes as (1) therapeutic misconceptions (the impact of perceptions from individuals that the purpose of research is for participant benefit rather than to produce generalisable knowledge) and (2) normalisation of deviations from good practice (where poor practice in cluster trials becomes normalised by perpetuation of continually expecting a lower level of evidence). Table 5 further describes these subthemes.

**Table 5:** Theme 4: unconscious biases

Subtheme	Explanation	Quote(s)
Therapeutic misconception (extrinsic barrier)	Therapeutic misconception, a term used to describe the phenomenon where individuals (usually patients) believe the purpose of research is for their direct benefit, rather than to produce generalisable knowledge,^29 30^ might also be a cause of bias in cluster trials. In cluster trials this materialises when healthcare workers (ie, those who identified participants for the trial) operate in a way so as to identify patients who would be more or less likely to benefit from the intervention their cluster had been assigned to, or patients more or less likely to agree to be included for the same reason.	“Whereas those who know it’s just usual care might be perhaps less less sort of enthusiastic at recruiting, you might get, you might get a, smaller numbers from them, they might forget to offer it to everybody, because they don't see much of a benefit for their GP surgery” (Professor of Behavioural Medicine, UK, 23yrs research experience) “if they're selectively inviting and they know their own allocation, UM, then they might very well invite the patients they think would, who would benefit most from the whatever it is, interventional or control” (Professor of Statistics, UK, 20yrs research experience) “They're really enthusiastic about the intervention they're less enthusiastic about not getting the bells and whistles” (Infectious Disease Clinical Researcher, Australia, 5yrs research experience)
	Some participants reflected on how while this might seriously undermine the study findings, it was almost never done out of malice.	“people behave differently and part of it is like most people are empathetic and they try to do best for everyone so the participant or patient or whatever would be in the scenario, so I don't think it’s done out of any malice, but I think they're trying to do what they think is best for that person. I don't have any great solutions, like it’s easy, I mean I don’t have a problem, the solution it’s yeah, it’s not easy.” (Director of clinical research group and Statistician, US, 20yrs research experience)
	Other participants reflected on how this bias might operate at the level of the healthcare professional.	“but you know if there’s if the intervention itself is something that may be, erm GP has contributed to maybe they are part of some sort of co-production process at the outset, or something like that and secretly they have, I’m invested in this, I think this is a great idea, now whether they're involved in the development or not, you know just the yeah, this is a good idea you know this might get government funding or something that future the trial does really well something like that, then yeah they're gonna be looking for the good patients to recruit into the trial erm and ones that they think actually whether it’s conscious or unconscious selection bias, erm there’s potentially a problem there” (Head of school, Australia, 15yrs research experience)
	While others reflected on how it might operate at the level of the research participant.	“people being more likely to agree [to] something if they sort of, you know, to agree to take part in the study if they think it might benefit them in some way. Um, so, if there’s an intervention that people think might be of help to them, they're more likely to maybe, if they know they’re going to get it and say, well you know I might as well try this intervention, Yes, I would like to be in the study. Erm whereas if you just say we want to collect your data and be part of a control group for a study but you're not going to get anything new or interesting or different.” (Post-doctoral statistician, UK, 5.5yrs research experience)
	Some participants were clearly already aware of this problem and had taken steps in their own trials to try to rectify it.	“We do a lot of training with the practice staff and the GPs kind of, you know to ensure that kind of the intervention is kind of you know, there is not kind of so much bias, and you know, that the GPs kind of understand that, you know there is this kind of risk of bias. … I think you know it would be good to have some sort of training of GPs etc, of the risks of cluster RCTs.” (Senior lecturer in translational research, UK, 9yrs research experience) “So that’s what we’re doing in one study is, you know, they’re recording the conversations that recruits have with patients, and then feeding back, sort of training to say, well actually you shouldn’t really use that type of language, because we, you know, we want to see if this intervention is beneficial. We don’t know It’s going to benefit them you know that kind of thing.” (Professor of Medical statistics, UK, 10yrs research experience)
Normalisation of deviance (extrinsic barrier)	Finally, we identified a theme whereby because conducting cluster trials in a way that they were at low risk of bias was so complicated, researchers were willing to compromise on rigour, in a way they would unlikely do in an individually randomised design. For example, one participant alluded to the hierarchy of evidence suggesting that a potentially biased cluster trial would have more rigour than a purely observational study.	“Sometimes this is the better study design to get the best evidence to answer the problem so therefore it’s, you know, it’s better than not doing the trial or doing a cohort or something instead.” (Infectious Disease Clinical Researcher, Australia, 5yrs research experience)
	Another participant expressed a very matter of fact acceptance of the problem.	“I don't think we've ever done a cluster randomised trial we haven't had imbalance, that’s kind of part of the course. I think we had fall off our chairs if we didn't get some balance.” (Group CEO of an institute, South Africa, 31yrs research experience)
	Others use the term ‘pragmatic’, but with reference to trial conduct rather than necessarily the research question.	“So I’m not someone who’s a trial purist who thinks that you must do perfect RCT’s in fact, I'd almost say that all of our trials have been pragmatic, which is mental model, which is sometimes code for messy.” (Infectious Disease Clinical Researcher, Australia, 5yrs research experience)
	Others, perhaps reflected on the somewhat paradoxical nature of this situation.	“And so if it’s on an important topic that we need evidence on, that’s still going to be the best level of evidence we have got on this topic. Um, but Yeah, sorry that’s a, that’s um a slight cop out.” (Post-doctoral statistician, UK, 5.5yrs research experience)
	Some participants talked about the need to compromise either on different types of bias.	“But then sometimes there’s no perfect, so there’s picking the least bad, the least imperfect, I guess.” (Professor of Medical statistics, UK, 10yrs research experience)
	Or on the need to accept a bias else otherwise not be able to conduct the study.	“Sometimes you have to compromise on one element of bias, to be able to sort of meet other important criteria like having enough people for the study to be meaningful in the first place is also you know, ethically important” (Professor of Behavioural Medicine, UK, 23yrs research experience)

## Discussion

### Summary

This semistructured interview study, including statisticians, clinical investigators and trial coordinators, with wide geographical and experience representation, should help inform how CRTs can be conducted so as they do not exhibit differential identification and recruitment of participants across the study arms.^5^ We identified four main themes: knowledge and misconceptions; alliances and collaborations; logistical and practical issues and unconscious biases. We also classified these themes into intrinsic and extrinsic factors.

Intrinsic factors, that is factors which could be viewed as under the control of the CRT research team, were identified as key contributing factors. First and foremost, knowledge related to biases around recruitment and identification of participants emerged as a key theme. While some participants were clearly very knowledgeable about these biases—many other participants lacked clear understanding of the issues or might have been aware of the issues but were confused about their importance or meaning. Collaborative team working could help sharing of this knowledge, but we also found that while some participants clearly preferred collaborative ways of working, others appeared to take a more tokenistic view of these collaborations.

We also identified the importance of extrinsic factors—factors which are not directly under the control of the researchers. For example, participants shared their experience of logistical and practical issues encountered when implementing recommended methods to mitigate bias, particularly the discourse between theory and practice, and the feasibility of implementing these recommendations. However, participants also offered their own grass-roots level solutions for successful implementation (eg, building into funding grants extra resources, or within trial monitoring to flag issues). Moreover, perhaps the most important finding of this work is the clear identification of the role unconscious biases have in perpetuating the biases in cluster trials. Similar to individually randomised trials, we identified a therapeutic misconception—here manifesting where those recruiting into the trial have a greater sense of a need to serve the patient rather than the research. We also identified a sense that a lower quality of evidence is acceptable in CRTs.

### What is known already

Multidisciplinary teams are key to the success of any type of randomised trial. Members of these teams play different complementary roles, inevitably with some perceived as having higher status than others. It is known that status and dynamics are important influences in productive collaborations.^20 21^ The statistician was historically considered a contributor rather than a partner in research—and this might mean they are perceived to have lower status.^22^ Furthermore, statisticians often work across many trials and so might not be as invested in the overall finding as the clinical partners. Communication and trust are also known to be important for effective collaborations, and this might come into play if the view of the statistician is unclearly communicated or given less prominence.^23^

We observed a general acceptance that the quality of evidence from CRTs might be low, but we were not able to identify why people considered it more acceptable in CRTs compared with other designs. However, in other settings, identified causes include simply following other poor examples; internal rationalisation (so the poor practice is known, but excuses to deviate from good practices are rationalised); and institutionalised (so the poor practice becomes so ingrained it is impossible not to follow).^24^ Moreover, it is even the case that sometimes poor practice becomes so prevalent that it becomes normalised.^24 25^ A classic example in healthcare is not washing hands at the appropriate times. These deviations from good practice are usually not ill-intended—so, for example, in the hand-washing case, they may arise from naivety or time pressure. The normalisation occurs when others observe this behaviour and the social pressure to conform with the good behaviour is then removed.^26^

There are already guidelines for prevention of identification and recruitment biases in CRTs.^5 7 27^ These include identification and recruitment by someone blind to the cluster treatment status. We identified that these recommendations were not always practical. Some additional practical suggestions include clear and objective eligibility criteria, the use of electronic records to identify all eligible participants (to avoid identification bias), restricting the use of CRTs with unblinded postrandomisation identification of participants only to settings where it is ethically appropriate to not need to directly recruit participants (ie, by waiver of consent); the inclusion of homogeneous clusters and piloting recruitment processes. Nonetheless, there were also suggestions for solutions that need to be dispelled as myths—so while covariate adjustment can help, it should not be viewed as a panacea; and these biases cannot be solved by restricted randomisation methods alone. Perhaps the most important practical suggestion came from the statisticians—when the CRT is at risk of these biases, an individually randomised trial is likely to be preferable.

### Strengths and limitations

This type of qualitative study is the first of its type in this area of research and has allowed us to gain a deeper perspective on the issues surrounding bias in CRTs. Recent advances in the use of electronic meetings (eg, Zoom) allowed the inclusion of participants from multiple countries. However, despite this, we had little representation from low-income and middle-income countries. Furthermore, the difficulties we encountered with recruitment resulted in a change to our planned recruitment strategy, and the snowballing approach very likely skewed our sample towards researchers with a particular interest in CRTs. Related to this, only 7 of the 24 participants had worked in CRTs for less than 5 years, and so we might not have captured the full spectrum of views from more junior researchers. All these reasons might mean that our findings are not necessarily generalisable to all CRTs.

Due to the intricate nature of the methods associated with CRTs, it was prudent for a statistician to conduct the interviews.^28^ Although the statistician who conducted the interviews had no qualitative experience, support was provided by the wider research team. Practice interviews were also conducted to gain experience in carrying out interviews prior to commencing the pilot phase of the study. Moreover, the nature of the interviews, trying to elicit reasons for poor practice, was sensitive. While we took steps to remove any direct criticism, researchers might not have felt able to provide uncensored views. For example, a few participants stated that they felt like they were being quizzed, although most did so in a good-natured way.

Various different levels of rapport developed throughout the interviews. Of note, some of the interviews were longer and the participants clearly passionate about the subject; others were shorter and with some sense that the participant perhaps did not share the same interest in the subject as our research team. Some of the participants were more relaxed than others and gave a sense of being able to talk openly. However, in other interviews, the participant occasionally had a more assertive role, clearly being very confident. With this variation evident across the interviews, a maintained realist approach was taken during the analysis, with all authors engaged to ensure we minimised our own biases when interpreting results. This important rapport acknowledgement relates to the complex status relationships which could be looked at in future work.

## Conclusions

There is an urgent need to ensure that CRTs are free from risks of bias to prevent serious threats to the validity of the findings. Mitigation measures are either not known, not practical or unconsciously subverted. More education might help, but so too might more collaborative working. Preventing subconscious bias at the time of participant recruitment and dispelling the myth that cluster trials produce a lower level of evidence are likely to require more of a change in culture. Some of the contributing factors are intrinsic to the study team, but others work at a higher level and are more extrinsic than internal. Given the findings of this study, it is clear that there is a need for appropriate and critical cluster expertise to enable a considered and practical approach to designing a CRT with selection bias at the forefront. These appropriate experts could be highly experienced researchers in either cluster methods work or applied researchers, including statisticians.

## Supplementary material

10.1136/bmjopen-2025-103091 online supplemental file 1

10.1136/bmjopen-2025-103091 online supplemental file 2

10.1136/bmjopen-2025-103091 online supplemental file 3

10.1136/bmjopen-2025-103091 online supplemental file 4

## Data Availability

No data are available.
