# Supplementary material for: Researchers’ views of risk of bias in cluster randomised trials: a qualitative interview study
Source: BMJ Open. 2025 Nov 5;15(11):e103091. doi: 10.1136/bmjopen-2025-103091 (PMC12593486; doi:10.1136/bmjopen-2025-103091)
Supplement: online supplemental file 3 [file bmjopen-15-11-s003.docx]

## Supplementary material 3: Illustrative case study to be sent to participants who have agreed to an interview

Title: Management of antipsychotic medication in adults with mild intellectual disabilities trial

This trial looked to test an intervention targeted at the patients (i.e., self-help material), to reduce the number of adults with learning disabilities who are prescribed antipsychotic medicine using a cluster randomised controlled trial. Due to the nature of the intervention this could not be blinded to all involved in this study (including the participant). The primary outcome was defined as the frequency of prescriptions for antipsychotic medication, measured on several occasions.

Forty GP practices were recruited to take part and were the unit of randomisation (1:1 ratio). This randomisation was completed by a clinical trials unit. The eligibility criteria for participants were as follows: adults aged 18 and over, who have been prescribed antipsychotics for more than 12 months, diagnosed with a mild intellectual disability, has not been admitted to hospital in the last 3 years, does not have a carer and does not have any diagnosed heart conditions.

The 40 GP practices, along with their GPs, were informed of their allocation group and began recruiting participants. Potential participants were identified by the GPs and invited to a consultation where they were assessed against the inclusion criteria. Those that were considered to be eligible were provided with information about the trial to consider taking part within the trial. After a 5-day consideration period, the patients attended a consultation with the same GP to determine if they would like to take part in the study. For those that agreed to take part, consent was taken and the GP then engaged the appropriate intervention or control condition as per the random allocation of that cluster.

**Baseline table for participant characteristics**

| **Characteristic** | **Control condition** | **Intervention condition** |
| --- | --- | --- |
|  | n=245 | n=302 |
| **Age, mean (SD)** | 45 (10.5) | 47 (11.4) |
| **Male, n (%)** | 105 (42.9) | 184 (60.9) |
| **Ethnicity, n (%)** |  |  |
| White | 80 (32.7) | 97 (32.1) |
| Mixed | 52 (21.2) | 62 (20.5) |
| Asian | 68 (27.8) | 89 (29.5) |
| Black, African or Caribbean | 39 (15.9) | 51 (16.9) |
| Other | 6 (2.4) | 3 (0.9) |
| **Length of diagnosis of MLD, years, mean (SD)** | 10 (4.5) | 15 (6.2) |
| **Years on antipsychotic medication, n (%)** |  |  |
| 1-2 years | 52 (21.2) | 55 (18.2) |
| 2-3 years | 110 (44.9) | 107 (35.4) |
| 3-4 years | 68 (27.8) | 97 (32.1) |
| More than 4 years | 15 (6.1) | 43 (14.2) |
| **Previously required specialist carers, n (%)** | 85 (34.7) | 132 (43.7) |
| **Received Cognitive behavioural therapy, n (%)** | 146 (59.6) | 209 (69.2) |
| **Number of antipsychotic prescriptions in the last 3 months, mean (SD)** | 3 (1.5) | 1 (0.9) |
