# Supplementary material for: Researchers’ views of risk of bias in cluster randomised trials: a qualitative interview study
Source: BMJ Open. 2025 Nov 5;15(11):e103091. doi: 10.1136/bmjopen-2025-103091 (PMC12593486; doi:10.1136/bmjopen-2025-103091)
Supplement: online supplemental file 4 [file bmjopen-15-11-s004.docx]

## Supplementary material 4: Topic guide for the semi-structured interviews

Study title

Exploring the implementation and conduct of cluster randomised controlled trials of individual-level interventions: a qualitative study using semi-structured interviews

Description of the topic guide

This is an outline of the anticipated interview structure; however, this will remain flexible and follow the direction of the interview itself. The points made in this topic guide are to ensure the interview covers key areas within the remit of the study objectives, whilst retaining flexibility, and not being limited to these areas.

Format of the topic guide

Topic guide structure

This topic guide is structured using the following sections:

- Introduction
- General questions
- Illustrative case study
- Recruitment (with focus on selection bias)
- Design justification
- Reflection questions
- Conclusion
- Feedback (if applicable)

The Introduction covers the practicalities of the interview, ensuring the participant understands the purpose of this interview and the study, and allowing for any questions before beginning the interview. The interview will then start by obtaining the interviewees’ characteristics, through general questions about their experience of being involved in cluster trials.

The illustrative case study, which will have been sent to the participant in advance of the interview, will then form the focus of the remaining questions. The questions concentrate on recruitment, selection bias and design justification aligning with the study objectives. Following this there is a reflection section.

Finally, in the conclusion of the interview, the interviewee will have a chance to add anything further and following this the main interview will be ended. For participants taking part in the pilot phase, they will then be asked to provide feedback on their experience of participating in the study. These feedback questions are focused on the recruitment process and the interview.

Section structure

The following structure will apply to each section:

- Bold questions or statements to be verbalised to the interviewee.
- Indented questions under the bold questions are prompt questions to be used when the discussion does not cover the specific areas of interest.

Introduction

The introduction will be completed using the following order:

- **To formally introduce myself, my name is Christina Easter and I am a part time PhD student and medical statistician.**
- **Thank you for agreeing to participate in this study and for returning the consent form and questionnaire. I just want to take this opportunity to ensure you are still happy to go ahead with this interview today.** (If the consent form has not been returned then ask for this to be sent before we formally start the interview.)
- **Firstly, are you familiar with using Zoom?** (If not, then just give a general overview of the buttons that they need to know, along with having to actively accept that the recording will take place and the live transcription.)
- If the interviewee does not have their camera on: **For this interview it would be appreciated that your camera is turned on during this, is it possible to turn your camera on for the duration of this interview?**
- **This interview process has been scheduled to take up to 60 minutes. We hope to be finished by XX (insert time the interview is anticipated to be completed by), is this still ok with your schedule? Is there another meeting you have to attend after this interview that I need to be mindful of?**
- **In brief the purpose of this study is to gain a more in-depth understanding of the challenges facing those conducting a cluster randomised trial. And so ultimately, we would like to help offer possible solutions and considerations for researchers, like yourself, planning to undertake and conduct cluster randomised trials.**
- **The semi-structure nature of this interview is not set in stone but there are some key areas which I would like to focus on.**
- **I would like to talk about your general experience of conducting cluster randomised trials, then turning our attention to the illustrative case study where subsequent questions will focus on this. However, for all questions, if you have insights from other trials please do feel free to mention them.**
- **Have you had chance to read the illustrative case study that was sent to you? If not, do not worry I will give you an overview of this case study during the interview.**
- **Throughout the interview you are very welcome to have this illustrative case study to hand to refer to.**
- **In this interview there are no correct or incorrect answers to the questions I will ask, this is purely to gather information on experiences and views, so you are free to say as little or as much as you would like.**
- **Also, I will be the only person who will have access to this recording and the other members of this research team will not be able to identify you from the interview transcription.**
- **If you are happy with everything, then I will start the recording.**

General questions on the interviewee’s experience of being involved in cluster trials

- **Could you very briefly talk me though your main roles when you have conducted cluster trials?**
- What is your current professional role?
- Are you currently part of a study unit, such as a clinical trials unit?
- What roles have you previously taken?
- How long have you been this type of researcher for?
  - How many years of experience do you have working on trials?
- How many cluster trials have you been involved in?
- What level of involvement have you previously had in conducting cluster trials?

Illustrative case study

**Let’s turn our attention to the illustrative case study.**

*(If interviewee has not read through this read through this case study)*

**Now before we focus on the questions on this case study, do you have any questions about this?**

Recruitment, selection bias and design justification questions

**For the following questions, I would like you to answer in relation to the illustrative case study. So please do have this to hand so that you can refer to it throughout the sections that follow.**

Recruitment of participants

- **In your own words, can you summarise how the participants recruited into the study?**
  - When were participants recruited?
  - Who recruited the participants into the study?
- **Can you talk through who might have known about the treatment allocation at the time of recruitment?**
  - Were the people who recruited the participants aware of the treatment allocation?
  - Was the participant aware of the treatment allocation?
  - Do you think this awareness of the treatment allocation by the GPs when recruiting participants is problematic?
  - Would it be problematic if the participant was aware of the treatment allocation at the time of recruitment?
- **Can you discuss how you might have modified this process with hindsight or with increased flexibility / resources?**
  - In your opinion, do you think that this approach for recruiting participants was optimal?
  - Do you think it was possible to conceal the treatment allocation from the person recruiting?
  - What would you consider modifying about this recruitment process?
  - What do you think the barriers were for implementing in this modified way?
  - What are your thoughts on if randomisation could not happen after recruiting all the participants into the study?
- **What are your thoughts about some of these suggestions?** (*mention these if not mentioned in the previous question*)

1. Participants should have been recruited prior to randomisation (optimal in this case as prevalent cases).
2. The treatments (intervention and control conditions) could have been concealed from participants and recruiters at the time of recruitment.
3. Participants could have been identified by somebody independent to the study team.

**Let’s now say that we have a study where the participants did not have the target condition already and so they are identified and recruited as the study progressed (incident cases).**

- **Can you talk about the methods that could be used to recruit participants?**
  - How would you approach the recruitment process in the most optimal way?
  - Are there challenges or difficulties with conducting the recruitment process in the most optimal way?
  - Do you think it is always feasible to undertake these methods?

Design justification

- **What do you think the primary drivers were for conducting a cluster randomised trial?**
  - Why do you think the study team chose to run this study as a cluster randomised trial?
  - What elements of this study lends itself to being conducted as a cluster randomised trial?
  - Are there other reasons (not specific to this illustrative case) which would justify using a cluster design?

Illustrative case study – Further information

**The trial has commenced and the data has been collected. A baseline table of participant characteristics has been reported split by treatment arm.**

**To just summarise the methods used for this study: The study was well randomised through use of a clinical trials unit and the GP practices and GPs at these practices were aware of the treatment allocation of the clusters at the time of recruitment. Where we are defining recruitment as when the patient was invited to take part in the study, consent was taken, therefore up until the point for which the treatment condition was instigated as per the treatment allocation of the cluster. All of these steps were conducted by the patients GP.**

- **What would have been the advantages of running the trial using individual randomisation instead of cluster randomisation?**
  - Could you discuss any differences in the characteristics of the participants in the two treatment arms, thinking about the importance of any differences?
  - What are the main implications if we see a baseline table that is unbalanced?
  - Would this sort of selection bias have arisen under individual randomisation?
- **What would have been the barriers to running the trial using individual randomisation?**
  - What part of the study design would cause concern for individual randomisation?
  - Could any of these barriers be overcome?

Reflection questions

- **Given the information provided on this case study, do you think that this was a good design choice for this study?**
- **In your own words how would you have designed this study in relation to recruitment and study design?**
- **Are there barriers to running the trial in the way you would want to do it?**
- **In your experience, what issues commonly arise surrounding recruitment and concealment of treatment conditions?**

Concluding questions

The conclusion will be completed using the following order:

- **Within this interview we have focused on recruitment of individual participants and the justification of undertaking a cluster randomised trial. I wondered if there was any further comments or information that you would like to add?**
- **Thank you for your time taking part in this interview, your insight and experience of undertaking a cluster randomised trial has been greatly appreciated.**
- **The next steps will be to ensure the transcribing is accurate and once completed this will be sent to you to have a look over to ensure you are happy with the accuracy of what was discussed in the interview.**
- If the interviewee has referred to a trial: **You have referred to a specific trial within this interview, are you happy with these aspects being included in the transcript that will be disclosed to the wider study team?**
- **Are you happy to be contacted and kept up-to-date on the findings of this study with respect to any publication that may arise from this qualitative study?**
- **If participant is part of the pilot phase, obtain feedback at this point using the questions outlined in the section below.**
- **Stop recording the interview.**

Pilot study feedback (Pilot participants to be asked this only)

The questions under the following sections will be asked to the interviewee as part of the feedback process.

Initial email and participant information sheet

- **Was there anything in the initial email sent to you that you feel could be improved?**
- **Was there anything that you feel did not come across well or could have been clearer in the email?**
- **Did the participant information sheet help you to make an informed decision?**
- **Were there further details that could be added to help improve the information sheet?**
- **Is there anything else you would like to comment on with respect to the email and participant information sheet?**

Content of the interview i.e. topic guide

- **How did you find the topic areas that were covered?**
- **Did you feel you wanted to talk about further areas of concern to yourself?**
- **How did you find the areas for which I focused on talking about?**
- **Is there anything that you feel should be taken out or added?**
- **Is there anything else you would like to comment on with respect to the interview process and areas of discussion?**
- **Lastly, if there is anything further after this interview process that you would like to feedback to me about then please do get in contact with me via email.**
