# Supplementary material for: Researchers’ views of risk of bias in cluster randomised trials: a qualitative interview study
Source: BMJ Open. 2025 Nov 5;15(11):e103091. doi: 10.1136/bmjopen-2025-103091 (PMC12593486; doi:10.1136/bmjopen-2025-103091)
Supplement: Supplementary file 5 [file bmjopen-2025-103091.reviewer_comments.pdf]

PEER REVIEW HISTORY

BMJ Open publishes all reviews undertaken for accepted manuscripts. Reviewers are asked to complete a checklist review form and are provided with free text boxes to elaborate on their assessment. These free text comments are reproduced below.

ARTICLE DETAILS

Title (Provisional)

Researchers' views of risk of bias in cluster randomised trials: a qualitative interview study

Authors

Easter, Christina L; Kristunas, Caroline; Greenfield, Sheila; hemming, karla

---

VERSION 1 - REVIEW

**Reviewer**	**1**
**Name**	**Rogers, Amy**
**Affiliation**	**University of Dundee, MEMO Research**
**Date**	**06-May-2025**
**COI**	**None**

Thank you for the opportunity to review this interesting paper that explores perceptions of bias in cluster randomised trials by researchers.

I have only a few minor comments:

1. Pg 7, line 53 - "(1) the discourse between theory and practice

(whereby recommendations for good practice simply did not work in the real-world)". Is discourse the right word here? My understanding is that "discourse between theory and practice " would mean a two-way interplay between the two, which may result in positive changes to both. Perhaps a more suitable alternative phrase to describe what interviewees referred to would be "gap between theory and practice" or "theory-practice disconnect".

2. Pg 9, line 24 "It is known that status and dynamics are important influences in productive collaborations." It would be interesting to know if you found any evidence of status at play in your results

3. Pg 11, line 10 "Various levels of rapport..." While this is interesting to reflect on, can you expand on whether this observed variation should be considered when interpreting your results?

**Reviewer**	**2**
**Name**	**Kelleher, Deirdre**
**Affiliation**	**Weill Cornell Medicine, Anesthesiology**
**Date**	**28-Sep-2025**
**COI**	**None**

Thank you for submitting your qualitative work investigating risk of bias in cluster RCTs. Your manuscript is very well written and clearly describes your interview process, rationale, and findings. The supplementary material supports the manuscript well. I have no suggestions for improvement and believe the study and manuscript are fully sound.

**Reviewer**	**3**
**Name**	**Sydenham, Emma**
**Affiliation**	**The Cochrane Collaboration, Cochrane Central Production Service**
**Date**	**06-Oct-2025**
**COI**	**None**

I enjoyed reading your study, and I think it will add to the literature that is sometimes used in the professional training of medical journal editors.

I think it was good to point out in the text that the interviewee based in Pakistan is actually from another country but is working in Pakistan. This is an important distinction to make, as it highlights the international nature of some research projects and the international mobility of some research staff. It is also why it is important for there to be international harmonisation of regulation and training. I am familiar with Pakistan's clinical trial regulations, which are harmonised with international standards. The comments of the interviewee in Table 2 are in fact at odds with the clinical trials regulations of the country in which they are working. I found this to be one of the most interesting points of your research findings. I do not think you need to make any changes to the text, I am just offering my reaction to one of the topics identified in your interview study.

The only thing I think you could amend is in Table 2: High-level knowledge, the Quote about GRADE. I think GRADE should be in capital letters as it refers to a specific framework. Not everyone reading the table will automatically understand what the quote is referring to if "GRADE criteria" is not identified as a specific framework.

In the second column, you could change the text to say "down-GRADE" rather than "down-grade" as currently written for further clarity.

VERSION 1 - AUTHOR RESPONSE

Reviewer 1

Comment 1

Pg 7, line 53 - "(1) the discourse between theory and practice (whereby recommendations for good practice simply did not work in the real-world)". Is discourse the right word here? My understanding is that "discourse between theory and practice " would mean a two-way interplay between the two, which may result in positive changes to both. Perhaps a more suitable alternative phrase to describe what interviewees referred to would be "gap between theory and practice" or "theory-practice disconnect".

Response 1

Thank you for picking up on this wording. We agree that the use of “discourse” is not quite right in this context. We have amended the wording to “(1) Theory-Practice disconnect”, which describes the scenario much better. [See Pg 8, paragraph 1]

Comment 2

Pg 9, line 24 "It is known that status and dynamics are important influences in productive collaborations." It would be interesting to know if you found any evidence of status at play in your results

Response 2

Thank you for highlighting this area of interest. Whilst there were no direct discussions on status and the role that plays in a research or study group, we did pick up on some hesitations from those participants that did not have as much experience in this area of research. On the other hand, the more established researchers in the field of cluster trials were very certain of themselves during discussions had within the interviews. In turn, these deduced findings were not specific *per say* to cluster trials themselves but rather the dementor of the participant with the possible complexity that a PhD student was conducting the interview. This certainly could be explored further, but with the perspective of different levels of researchers to understand this complex relationship. We have added in a sentence to highlight that this would be of interest in future work.

“This important rapport acknowledgement relates to the complex status relationships which could be looked at in future work.”

Comment 3

Pg 11, line 10 "Various levels of rapport..." While this is interesting to reflect on, can you expand on whether this observed variation should be considered when interpreting your results?

Response 3

Thank you for your observation. Whilst the interviewer lead on the analysis of the interview content, a realist approach was maintained with input from the other authors grounding the results such that any "biases" that the interviewee had was not significantly reflected throughout the findings. However, this is a consideration especially with the natural fact of human interactions and individual personalities and approaches influence the rapport creating during hour long interviews. We have added the following text to the manuscript on Page 11, Paragraph 2:

"With this variation evident across the interviews, a maintained realist approach was taken during the analysis, with all authors engaged to ensure we minimised our own biases when interpreting results."

Reviewer 2

Thank you for taking the time to review this manuscript.

Reviewer 3

<u>Comment 1</u>

I think it was good to point out in the text that the interviewee based in Pakistan is actually from another country but is working in Pakistan. This is an important distinction to make, as it highlights the international nature of some research projects and the international mobility of some research staff. It is also why it is important for there to be international harmonisation of regulation and training. I am familiar with Pakistan's clinical trial regulations, which are harmonised with international standards. The comments of the interviewee in Table 2 are in fact at odds with the clinical trials regulations of the country in which they are working. I found this to be one of the most interesting points of your research findings. I do not think you need to make any changes to the text, I am just offering my reaction to one of the topics identified in your interview study.

<u>Response 1</u>

Thank you for taking the time to detail this specific observation. We have not made any further amendments to the manuscript given this comment.

<u>Comment 2</u>

The only thing I think you could amend is in Table 2: High-level knowledge, the Quote about GRADE. I think GRADE should be in capital letters as it refers to a specific framework. Not everyone reading the table will automatically understand what the quote is referring to if "GRADE criteria" is not identified as a specific framework.

<u>Response 2</u>

Thank you for bringing this to our attention. We agree that GRADE should be written in capital letters to note that it refers to a specific framework. Additionally, we have included further clarification for this acronym and what we are referring to specifically in Table 2, Column 3, Row 1.

Comment 3

In the second column, you could change the text to say "down-GRADE" rather than "down-grade" as currently written for further clarity.

Response 3

Thank you for this suggestion. This amendment would certainly provide further clarity by changing the format of this word. We have adjusted the text accordingly. [See Table 2, Column 3, Row 1]
