# Supplementary material for: Researchers’ views of risk of bias in cluster randomised trials: a qualitative interview study
Source: BMJ Open. 2025 Nov 5;15(11):e103091. doi: 10.1136/bmjopen-2025-103091 (PMC12593486; doi:10.1136/bmjopen-2025-103091)
Supplement: online supplemental file 1 [file bmjopen-15-11-s001.docx]

## Supplementary material 1: Updated main phase email

**Subject:** Exploring the implementation and conduct of cluster randomised controlled trials: a qualitative study using semi-structured interviews

Dear Professor/Dr./Mr./Ms **[Insert corresponding authors name here]**

My name is Christina Easter, I am a PhD student at The University of Birmingham, working with Professor Karla Hemming. I am currently conducting a qualitative study exploring the implementation and conduct of cluster randomised trials of interventions that have been administered at the individual-level (i.e. the intervention delivered to the participant). This study aims to explore the experiences and challenges faced by principal investigators, trialists and statisticians when planning and undertaking a cluster randomised controlled trial.

You have been identified as somebody who has been involved in a cluster randomised trial. Therefore, I would like to formally invite you to take part in our study, by participating in a short interview over Zoom. Your participation in this study will help us achieve a deeper understanding of the methodological and logistical issues for which professionals, like yourself, face when planning and conducting a cluster trial. Together we would like to identify the areas which are challenging, to aid future researchers in negotiating through such challenges with more ease, ultimately providing further guidance to support investigators undertaking cluster trials.

An illustrative case study will be the focus of the interview. Further information about this study can be found in the attached participant information sheet, which I would encourage you to read before agreeing to participate. If you would be interested in participating in this study, then please let me know, ideally within two weeks of receiving this email, so that we can arrange a convenient time to meet.

Our intention is to explore the views of a range of stakeholders including principal investigators, trialists and statisticians to gain different perspectives. It would therefore be greatly appreciated if you could forward this invitation on to any members within the research team who might also be interested in talking to me; or forward their contact details to me.

If you would like to take part in our study, you will receive a £50 Amazon voucher upon completion of the interview.

If you have any further questions about this research please do not hesitate to contact me.

Yours sincerely,

Christina Easter

PhD Student

Medical Statistician

Institute of Public Health Research

The University of Birmingham, UK

Email: [c.l.easter@bham.ac.uk](mailto:c.l.easter@bham.ac.uk)

Student email: [cxe938@student.bham.ac.uk](mailto:cxe938@student.bham.ac.uk)
