# Supplementary material for: Researchers’ views of risk of bias in cluster randomised trials: a qualitative interview study
Source: BMJ Open. 2025 Nov 5;15(11):e103091. doi: 10.1136/bmjopen-2025-103091 (PMC12593486; doi:10.1136/bmjopen-2025-103091)
Supplement: online supplemental file 2 [file bmjopen-15-11-s002.docx]

## Supplementary material 2: Targeted approach email

**Subject:** Exploring the implementation and conduct of cluster randomised controlled trials: a qualitative study using semi-structured interviews

Dear Professor/Dr./Mr./Ms **[Insert initial contact name here]**

We are currently conducting a qualitative study exploring the implementation and conduct of cluster randomised trials. This study aims to explore the challenges faced by those planning and undertaking a cluster trial. Together we would like to identify the areas which are challenging, to aid future researchers negotiate these challenges, ultimately providing further guidance to support investigators undertaking cluster trials.

We would like to invite principal investigators, trialists and statisticians that have been involved in cluster trials to take part in a short interview over Zoom. We are currently trying to identify statisticians, trialists, and principal investigators who have been involved in cluster trials who might be interested in taking part in this study.

We are therefore contacting you to ask if you know of any suitable participants. We would then ask you to either forward their contact details to us, or to forward this invitation onwards. I have attached the participant information sheet for more information about the study.

If you have any questions about this study please do get in contact with us.

Yours sincerely,

Christina Easter and Karla Hemming

Christina Easter

PhD Student

Medical Statistician

Institute of Public Health Research

The University of Birmingham, UK

Email: [c.l.easter@bham.ac.uk](mailto:c.l.easter@bham.ac.uk)

Student email: [cxe938@student.bham.ac.uk](mailto:cxe938@student.bham.ac.uk)

Karla Hemming

Professor of biostatistics

The University of Birmingham, UK

Email: [k.hemming@bham.ac.uk](mailto:k.hemming@bham.ac.uk)
